# Depression and Resting Masticatory Muscle Activity

**DOI:** 10.3390/jcm9041097

**Published:** 2020-04-12

**Authors:** Grzegorz Zieliński, Aleksandra Byś, Michał Ginszt, Michał Baszczowski, Jacek Szkutnik, Piotr Majcher, Piotr Gawda

**Affiliations:** 1 Department of Sports Medicine, Medical University of Lublin, 20-093 Lublin, Poland; grzegorz.zielinski.um@gmail.com (G.Z.); bysaleksandra@gmail.com (A.B.); piotr.gawda@umlub.pl (P.G.); 2 Department of Rehabilitation and Physiotherapy, Medical University of Lublin, 20-093 Lublin, Poland; m.baszczowski@gmail.com (M.B.); piotr.majcher@umlub.pl (P.M.); 3 Department of Functional Masticatory Disorders, Medical University of Lublin, 20-081 Lublin, Poland; jacek.szkutnik@umlub.pl

**Keywords:** depression, electromyography, masseter muscle, anterior temporal muscles

## Abstract

Background: The aim of this study was to determine the influence of moderate depression determined based on Research Diagnostic Criteria for Temporomandibular Disorders (RDC/TMDs) Axis II on the bioelectrical resting activity of temporal muscles and masseter muscles. Methods: The research participants were 68 healthy adult women. Of these, 46 people h (mean age: 22 ± 1 year) who had no temporomandibular disorders were selected for the research. They were divided based on results from RDC/TMDs (Axis II: scale’s measurement) into the study group with a moderate level of depression (23 people), rest of participants without symptoms of depression were classified to control group (23 people). The resting activity of temporal muscles and masseter muscles was examined by using BioEMGIII electromyograph. Two statistical analyses were used: Shapiro–Wilk test and Mann–Whitney U test. Results: Despite higher mean tensions of temporal muscles and masseter muscles in the group with depression, results findings were not statistically significant (*p* > 0.05). Conclusions: Moderate depression determined based on the RDC/TMDs II axis questionnaire is not related to the resting activity of selected masticatory muscles. Further research should be continued on a larger group of respondents in order to establish the relationship between psychological factors and bioelectrical parameters of the masticatory muscles.

## 1. Introduction

Depression is a common, serious, and recurring disorder that is associated with a decrease in quality of life, impaired social functioning, increased morbidity, and premature mortality [1]. World Health Organization (WHO) recognized depression as the fourth cause of disability and assumed that by 2020 it will have become the second main cause of it [2]. Depression, along with other psychological factors, such as anxiety or catastrophization, may play an important role in the development of chronic pain. In patients diagnosed with the occurrence of dysfunctions characterized by the presence of chronic pain, the coexistence of depressive symptoms is often observed [3]. It is estimated that 30–54% of people with chronic pain are also depressed [4]. Depression is also associated with disorders within the stomatognathic system. The incidence of depression in people diagnosed with temporomandibular disorders (TMDs) is 30.4%, while in the Polish population it is estimated at 15.8% [5,6].

The Research Diagnostic Criteria for Temporomandibular Disorders, (RDC/TMDs) allow biaxial assessment of the masticatory system disorders ensuring both structural and psychosocial/behavioral dysfunctions [7]. Axis I is based on the evaluation of the subject and clinical examination. Axis II of the RDC/TMDs form is based on three instruments: graduated chronic pain score (GCPS), Symptoms CheckList-90R (SCL-DEP), and somatic (SCL-SOM) depression scale. Axis II of the RDC/TMDs is screening due to the low sensitivity of the patient’s actual mental state. Based on the results of the last two scales, patients can be diagnosed in terms of severity of depression and somatization [8]. Taking into account the psychological factors that may influence the occurrence of chronic pain within the muscular system of the masticatory system in the assessment of TMDs is an important element influencing the selection of appropriate therapeutic methods adapted to the clinical condition of the patient. Diagnostics of temporomandibular disorders can be extended with computed tomography, magnetic resonance imaging, or X-ray imaging. Nonetheless, these methods are hard to reach, expensive, and limited in the assessment of clinical symptoms of TMDs [9]. Other examinations used in diagnostics of TMDs are mandibular kinesiography (MKG), ultrasonography, and electromyography (EMG) [10]. EMG is used to record muscle activity by detecting and recording the electrical potential generated by electrical or neural stimulation [9,11]. In contrast to needle-type EMG, surface electromyography (sEMG) is a noninvasive method which analyzes the total muscle activity without any pain [12]. This examination is considered the most valid and reliable method for the evaluation of muscle function and efficiency [13].

Numerous clinical studies prove that depression has a substantial effect on the stomatognathic system, including masticatory muscle activity, which can lead to temporomandibular disorders [5,6]. Moreover, an increase in the bioelectrical activity of masseter muscles was observed in the subjects with depression symptoms [14]. Therefore, the aim of this study was to determine the influence of depression determined on the basis of RDC/TMDs Axis II on the bioelectrical resting activity of temporal and masseter muscles.

## 2. Materials and Methods

### 2.1. Study Population

The study was carried out in accordance with the recommendations of the Helsinki Declaration and with the consent of the Bioethical Commission of the Medical University of Lublin (KE-0254/73/2017). The examined patients were informed about the research goals, they were aware of the possibility of resigning at any time. A written permission was obtained from all the respondents who participated in the study.

Sixty-eight women applied for the examination. The inclusion criteria used in the study were: age range 18–30 years, female gender, good or very good general health status according to the RDC/TMD questionnaire, no trauma of surgical intervention within the skull, without bite open, with full dentition, maintaining four support zones in dental arches, without inflammation in the oral cavity. The following exclusion criteria were used: functional disorders of the masticatory organ determined on the basis of the clinical RDC/TMD examination, pain in the craniofacial region and the cervical spine, possession of an orthodontic appliance, people with a malocclusion, neurological disorders, as well as injuries and surgical procedures within the head and cervical spine, and pregnancy.

After applying the above criteria, 46 women were qualified for the study (mean age: 22 ± 1 year). The RDC/TMD form (Axis II: SCAL Measurement) was used to measure the level of depression. The assessment of depression based on this scale allows for referral of a patient to a specialist clinic. Based on the results from the scale, the subjects were divided into two groups. The RDC/TMD questionnaire (Axis II: SCAL Measurement) was administered by a dentist trained in working with the questionnaire.

The control group consisted of 23 women (mean age: 22 ± 1 year) who had a level of depression of 0. It was a generally healthy group, meeting both the exclusion and inclusion criteria. Twenty-three women with moderate depression were present in the test group (mean age: 22 ± 1 year). It was a group meeting the exclusion and inclusion criteria. In which, after analyzing the Axis II of the RDC/TMD screening questionnaire, the level of depression was defined as moderate. The control and study groups were recruited among students of the Medical University of Lublin.

The point values classifying to a given group were determined based on the procedure developed by Ohrbach et al. (Norm: <0.535, moderate: 0.535 to <1.115) [15]. The examination was conducted in the morning hours (8–11 a.m.) to reduce the effect of daily muscle bioelectrical variation.

### 2.2. Electromyographic Measurements

The electromyographic examination was carried out in a dental chair. The patients were in a sitting position, with the body perpendicular to the ground, the head resting on the headrest of the chair and lower limbs upright and arranged parallel to each other. The height of the headrest was adjusted individually to set the head, neck, and torso of the subjects in a straight line. Before placing the surface electrodes, the skin of the subjects was cleaned with 90% ethanol solution. Next, surface electrodes (Ag/AgCl with a diameter of 30 mm and a conductive surface of 16 mm—SORIMEX, Toruń, Poland) were placed in accordance with the course of the muscle fibers of the temporal anterior torso (TA) and the surface part of the masseter muscle (MM) according to the SENIAM (surface EMG for non-invasive assessment of muscles) program guidelines [16]. The arrangement of the electrodes symmetrically on the skin covering the examined muscles on both sides in accordance with the course of muscle fibers was preceded by palpation of the muscles during mandibular movements. The electrodes on the surface of the masseter muscle were located along the line from the mandible angle to the inferior border of the zygomatic bone. The electrodes on the anterior part of the temporal muscle were arranged along a perpendicular line from the superior border of the zygomatic bone to a cranial bone (in the projection of the sphenoid bone). Symmetrically two electrodes were glued to the middle of each muscle. The edges of the electrodes covering the skin above a given muscle were in contact with each other to maintain a constant spacing between the electrodes. The reference electrode was placed on the forehead [17]. The electrode was placed in the center of the frontal bone between the frontal eminences and superciliary arches. The study involved an eight-channel BioEMG IIITM surface electromyography apparatus with BioPak Measurement System (BioResearch Associates, Inc., Milwaukee, WI, USA). Before the examination, an interference test was performed. Muscle activity in the masticatory system (TA, MM) was recorded in the resting position of the mandible for 10 s. During the examination, the subjects were instructed not to swallow saliva, to open their mouths, and to close their eyes.

The electromyographic signals obtained during the test were standardly amplified and purified from 99% of the noise scale on a linear scale using the BioPak digital NoiseBuster filter. Automatic processing of the electromyographic signal, based on the root mean square (RMS) calculation in the BioPak program, allowed us to obtain average measurement values, which were then used to analyze muscle activity.

Data analysis was carried out according to the following criteria:

(1) The comparison of the bioelectrical mean values of the anterior parts of the temporal muscle in the resting state between the control and test group.

(2) The comparison of the average bioelectrical values of superficial masseter muscles in the resting state between the control and test group.

### 2.3. Statistical Analysis

The comparison of bioelectric data was developed statistically using the IBM SPSS STATISTICS 21 program. The Shapiro–Wilk test and the Kolmogorov–Smirnov test (with the Lilliefors correction) were applied first in order to verify the normality of the distribution. When the distribution was close to normal, the Student *t*-test was used, and when it was normal, the non-parametric Mann–Whitney U test was used. The level of significance was determined at 0.05 (5%).

## 3. Results

There were no significant differences in age (*p* = 0.658) and range of mandibular movements during maximum mouth opening (*p* = 0.877) between the test group (with depression) and controls (Table 1 and Table 2). The mean resting bioelectrical activity of the frontal temporal muscle in the test group was 4.54 μV/s, whereas that in the control group was 2.51 μV/s. However, these results were not statistically significant *p* = 0.792 (Table 3). The mean resting bioelectrical activity of the front parts of masseter muscles in the test group was 1.64 μV/s, while in the control group it was 1.51 μV/s. These results did not reach the assumed level of significance *p* = 0.767 (Table 4).

## 4. Discussion

The aim of this study was to determine the influence of moderate depression determined on the basis of RDC/TMDs Axis II on the bioelectrical resting activity of temporal muscles and masseter muscles. The average bioelectrical voltage on the anterior temporal part was higher in the study group by 2.03 μV/s; however, the result was not statistically significant *p* = 0.792. The average bioelectric voltage recorded on the masseter muscle was higher in the study group by 0.13 μV/s. These results were not statistically significant *p* = 0.767. There were also no differences in age between groups that could affect the results of the electromyographic examination. Moreover, the range of mandibular movements during maximum active mouth opening, obtained in the RDC/TMD examination, was also not significantly different between the two groups, which also reduces the effect of mandible movement range on the bioelectrical activity of the masticatory muscles. The selection of the study group in terms of age is related to the fact that mental disorders are frequent in the selected group. The study shows that mental health problems are prevalent in the younger population [18]. A report from 2014 concerning the well-being of young people from the UK proved that one in five young adults had symptoms of anxiety or depression [19]. Additionally, most mental disorders including dysthymia, major and minor depression, and anxiety are more frequent in women than in men [20]. Defining the level of the disorder was guided by the fact that a small part of the literature on the subject differentiates the degree of depression [21]. Determining the level is important to determine whether any depression will affect voltage changes. A moderate level of depression was chosen due to the lack of literature on subjects examining the disease in question and its effect on the bioelectric parameters of masticatory muscles.

The results of the presented study are in accordance with the results presented by Marchesi et al., who studied the incidence of migraines and headache tensions in people with depressive disorders [22]. They did not observe any significant differences in the prevalence of migraine and headaches associated with muscle tones in patients with major depression, bipolar depression, and dysthymic disorders [22]. A review of the literature conducted by Wieckiewicz et al. on the subject of mental status as a common factor of masticatory muscle pain shows that the causal connection between mental states and masseter muscle pains is still not clearly stated and explained [21]. This is consistent with the results of the presented study—no statistically significant greater masticatory muscle activity (TA, MM) was observed in subjects with depressive disorders. On the other hand, Stocka et al. reported an increase in the bioelectrical activity of masseter muscles in maximal voluntary clench in the group of subjects with depression symptoms [14]. However, the above examination was carried out only during teeth clenching and did not include the resting activity in contrast to the presented work.

Tae-Jin Song et al. showed that anxiety and depression were associated with the exacerbation of headache symptoms among patients with tension headaches [23]. According to a study by Bonjardim et al. carried out with the use of the Hospital Anxiety and Depression Scale (HADS), anxiety and depression affect the subjective symptoms of TMDs, but only anxiety is correlated with clinical symptoms, such as muscle tone [24]. This confirms our research on the impact of depression on the masticatory muscle tone disorders, while in terms of the impact of anxiety on temporomandibular disorders there is no unambiguous position. Moreover, studies by Calixtre et al. confirm the lack of dependence between depression and temporomandibular disorders, however, they exclude the correlation of anxiety and the occurrence of TMD symptoms [25]. Studies by Bonjardim et al. [24] and Calixtre et al. [25] indicate that despite the use of different scales of anxiety and depression, the conclusion regarding the impact of depression on TMDs is the same. However, in terms of the impact of anxiety, no unambiguous position was identified, further studies are recommended to confirm these assumptions.

To sum up, moderate depression determined based on the RDC/TMDs II axis questionnaire is not related to the resting activity of selected masticatory muscles. These observations may exclude the participation of the temporal muscles and the masseter in the etiology of tension headaches or bruxism in people with the discussed level of depression. This can speed up the diagnosis and exclude psychosomatic disorders as a cause of muscle tone in the muscles of the masticatory system. Future research should be continued on a larger, age-diverse group. It is also worth conducting studies at a higher level of depression to establish the relationship between psychological factors and bioelectric parameters of masticatory muscles.

The presented study has several limitations. Firstly, the diagnostics criteria for TMDs were changed to DC/TMDs in 2014; however, in the presented study, the previous version was used. There is no validated Polish version of the DC/TMDs so far, therefore, the RDC/TMDs was used. Secondly, Axis II of the RDC-TMD is a screening tool to evaluate the presence of the psychosocial and behavioral dysfunctions in patients with temporomandibular disorders. In this study, it was used for people without signs and symptoms of TMDs. However, Axis II of the RDC-TMDs includes a depression scale adapted from the SCL-90, which is widely used to measure subjective psychopathology. Moreover, the screening efficiency of Axis II of the RDC-TMDs is quite good, at 87% sensitivity (normal vs, moderate/severe depressive symptoms) [26]. Thus, the RDC/TMDs depression scale has good psychometric properties for screening for psychological distress. Thirdly, the study sample consists of a small group with the participation of only women, with a low average age index. Hence, future research should include relevant groups from a larger population with an expanded age range.

## 5. Conclusions

Moderate depression determined based on the RDC/TMDs II axis questionnaire is not related to the resting activity of selected masticatory muscles. Further research should be continued on a larger group of respondents in order to establish the relationship between psychological factors and bioelectrical parameters of the masticatory muscles.

## Figures and Tables

**Table 1:** Average age (±SD) in the group without depression (control group) and the group with depression.

Group	*n*	Mean Age (years)	SD	*t*	*p*
Control Group	23	22	0.86	0.455	0.658
Group with Depression	23	22	1.18		

**Table 2:** The comparison of mean maximum mouth opening (MMO) between the group without depression (control group) and the group with depression.

Group	*n*	Mean MMO (mm)	SD	*Z*	*p*
Control Group	23	50.57	5.66	−0.154	0.877
Group with Depression	23	51.30	6.49		

**Table 3:** The comparison of mean activity of temporal muscle (TA) in rest between the group without depression (control group) and the group with depression.

Group	*n*	Mean sEMG Activity (µV/s)	Median sEMG Activity (µV/s)	SD	*p*
Control Group	23	2.51	2.45	1.12	0.792
Group with Depression	23	4.54	2.57	9.87	

sEMG: surface electromyography.

**Table 4:** The comparison of mean activity of masseter muscle (MM) in rest between the group without depression (control group) and the group with depression.

Group	*n*	Mean sEMG Activity (µV/s)	Median sEMG Activity (µV/s)	SD	*p*
Control Group	23	1.51	1.40	0.53	0.767
Group with Depression	23	1.64	1.51	0.83	
